# Supplementary material for: Workplace Integrated Safety and Health Program Uptake in Nursing Homes: Associations with Ownership
Source: Int J Environ Res Public Health. 2021 Oct 28;18(21):11313. doi: 10.3390/ijerph182111313 (PMC8583467; doi:10.3390/ijerph182111313)
Supplement: Supplementary file 1 [file ijerph-18-11313-s001.zip › Predicting WISH Supplementary Materials Tables.pdf]

Supplementary Materials

Figures S1A–S1E. Distribution of WISH domain scores.

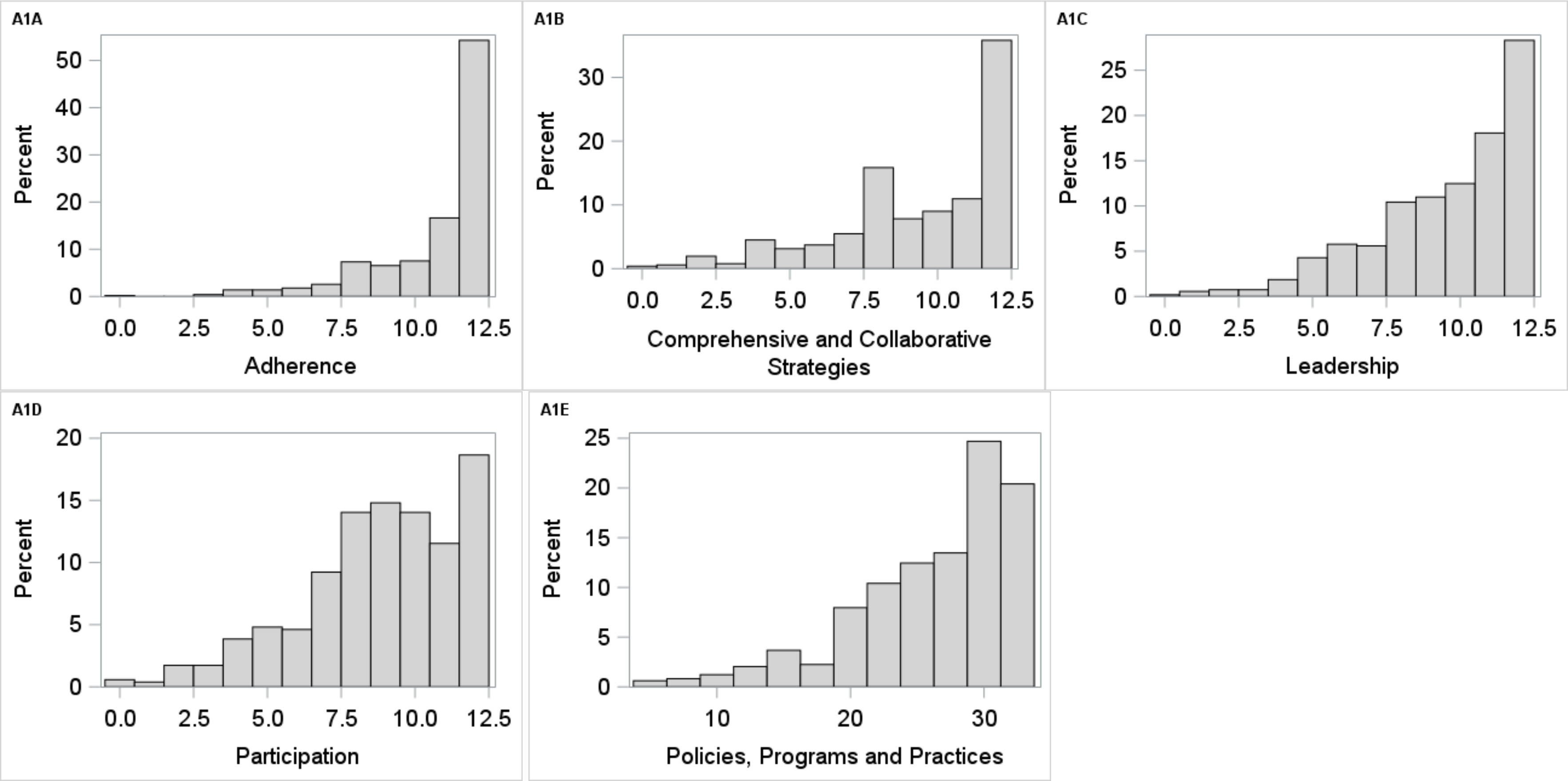

Figures S2A, S2B. Distribution of WISH domain scores by ownership status. CompStrategy= Comprehensive and Collaborative Strategies; PPP=Policies, Programs, and Pratices. PPP re-scaled to 0-12.

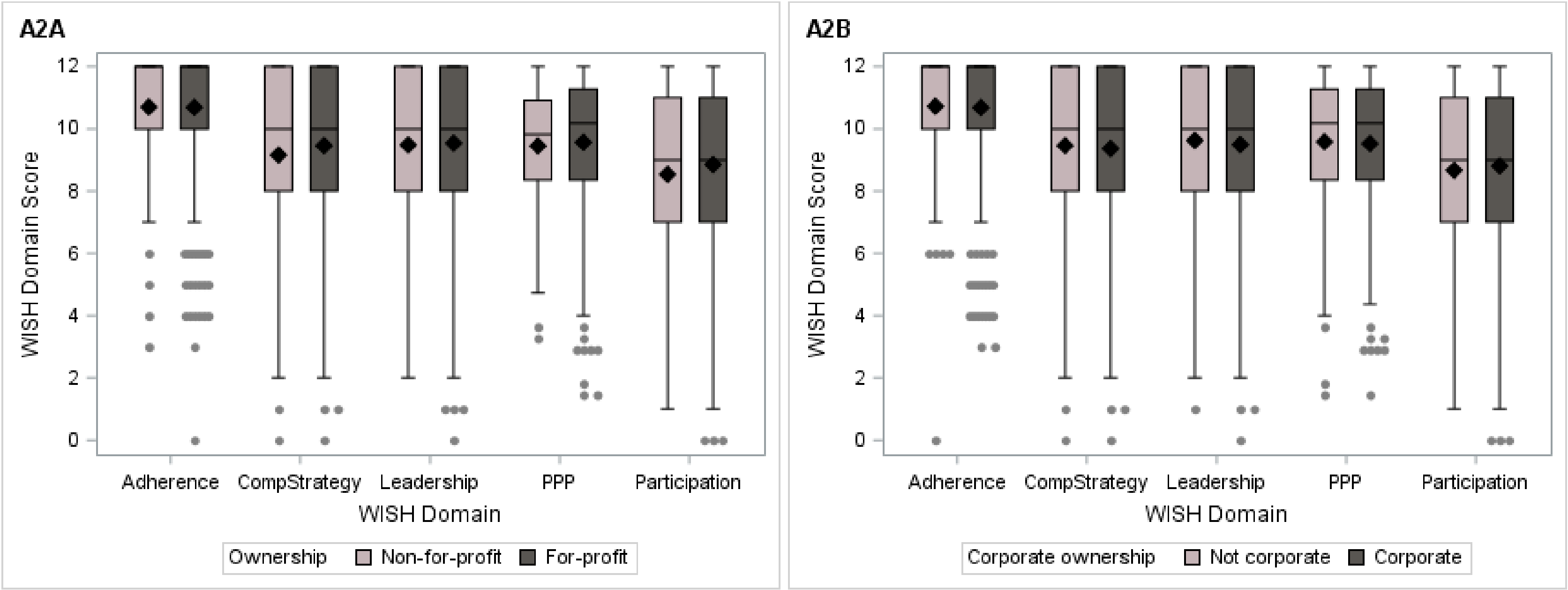

Table S1: Spearman Correlation Coefficients between WISH Domains

**Variable**	Adherence	Comprehensive and Collaborative Strategies	Leadership	Participation	Policies, Programs and Practices
Adherence	1.0	0.62	0.59	0.55	0.70
Comprehensive and Collaborative Strategies		1.0	0.69	0.68	0.78
Leadership			1.0	0.70	0.69
Participation				1.0	0.67
Policies, Programs and Practices					1.0

Table S2: Associations between for-profit nursing home ownership and being in the bottom 25% percentile of WISH domain

	Model 1			Model 2			Model 3		
**Variable**	**Odds Ratio**	**95% CI**	**p-value**	**Odds Ratio**	**95% CI**	**p-value**	**Odds Ratio**	**95% CI**	**p-value**
Adherence	0.95	[0.55, 1.65]	0.8506	0.98	[0.54, 1.77]	0.9364	0.81	[0.43, 1.51]	0.4997
Comprehensive and Collaborative Strategies	0.74	[0.43, 1.30]	0.3009	0.71	[0.39, 1.29]	0.2612	0.66	[0.34, 1.26]	0.2068
Leadership	1.34	[0.76, 2.37]	0.3123	1.35	[0.73, 2.50]	0.3394	1.05	[0.54, 2.03]	0.8854
Participation	0.78	[0.47, 1.27]	0.3119	0.79	[0.47, 1.34]	0.3784	0.67	[0.38, 1.18]	0.1631
Policies, Programs and Practices	0.98	[0.57, 1.71]	0.9511	0.96	[0.53, 1.73]	0.8858	0.78	[0.42, 1.48]	0.4511

Table S3: Associations between corporate nursing home ownership and being in the bottom 25% percentile of WISH domain

	Model 1			Model 2			Model 3		
**Variable**	**Odds Ratio**	**95% CI**	**p-value**	**Odds Ratio**	**95% CI**	**p-value**	**Odds Ratio**	**95% CI**	**p-value**
Adherence	1.18	[0.71, 1.97]	0.5176	1.23	[0.73, 2.06]	0.4412	1.25	[0.74, 2.11]	0.4078
Comprehensive and Collaborative Strategies	1.08	[0.65, 1.80]	0.7667	1.10	[0.65, 1.87]	0.7109	1.07	[0.63, 1.83]	0.7903
Leadership	1.24	[0.74, 2.09]	0.413	1.26	[0.73, 2.16]	0.4037	1.24	[0.72, 2.14]	0.446
Participation	0.76	[0.49, 1.19]	0.2297	0.73	[0.46, 1.16]	0.1887	0.74	[0.47, 1.18]	0.2119
Policies, Programmes and Practices	1.00	[0.61, 1.64]	0.9911	1.00	[0.60, 1.67]	0.9911	1.02	[0.61, 1.70]	0.9435

Model 1: Adjusted for survey wave and state
Model 2: Adjusted for survey wave, state, number of beds, occupancy rate, percent of residents who are Medicaid recipients
Model 3: Adjusted for survey wave, state, number of beds, occupancy rate, percent of residents who are Medicaid recipients, staffing patterns

Table S4: Associations between for-profit nursing home ownership and each WISH domain: sensitivity analysis for WISH cut-points

	**Bottom 10%**			**Bottom 25%**			**Bottom 33%**		
**Variable**	**Odds Ratio**	**95% CI**	**p-value**	**Odds Ratio**	**95% CI**	**p-value**	**Odds Ratio**	**95% CI**	**p-value**
Adherence	0.89	[0.35, 2.28]	0.807	0.98	[0.54, 1.77]	0.936	1.03	[0.60, 1.77]	0.923
Comprehensive and Collaborative Strategies	0.61	[0.26, 1.45]	0.260	0.71	[0.39, 1.29]	0.261	0.66	[0.40, 1.08]	0.101
Leadership	1.38	[0.57, 3.30]	0.475	1.35	[0.73, 2.50]	0.339	0.95	[0.57, 1.59]	0.848
Participation	0.63	[0.27, 1.47]	0.286	0.79	[0.47, 1.34]	0.378	0.79	[0.47, 1.34]	0.378
Policies, Programs and Practices	0.94	[0.41, 2.18]	0.892	0.96	[0.53, 1.73]	0.886	1.01	[0.60, 1.71]	0.961

Table S5: Associations between corporate nursing home ownership and each WISH domain: sensitivity analysis for WISH cut-points

	**Bottom 10%**			**Bottom 25%**			**Bottom 33%**		
**Variable**	**Odds Ratio**	**95% CI**	**p-value**	**Odds Ratio**	**95% CI**	**p-value**	**Odds Ratio**	**95% CI**	**p-value**
Adherence	1.28	[0.56, 2.95]	0.556	1.23	[0.73, 2.06]	0.4412	1.00	[0.60, 1.67]	0.983
Comprehensive and Collaborative Strategies	0.96	[0.45, 2.06]	0.922	1.10	[0.65, 1.87]	0.7109	0.99	[0.63, 1.57]	0.910
Leadership	1.72	[0.73, 4.03]	0.213	1.26	[0.73, 2.16]	0.4037	0.98	[0.63, 1.50]	0.639
Participation	2.10	[0.84, 5.24]	0.111	0.73	[0.46, 1.16]	0.1887	0.90	[0.58, 1.40]	0.189
Policies, Programs and Practices	1.23	[0.59, 2.58]	0.578	1.00	[0.60, 1.67]	0.9911	0.73	[0.46, 1.16]	0.610

Adjusted for survey wave, state, number of beds, occupancy rate, percent of residents who are Medicaid recipients

Table S6: Associations between for-profit nursing home ownership and each WISH domain: sensitivity analysis comparing top to bottom quartile. Results presented show adjusted odds of being in the lowest vs. highest WISH quartile for for-profit vs. not-for-profit nursing homes.

**Variable**	**Odds Ratio**	**95% CI**	**p-value**
Adherence	0.90	[0.48, 1.67]	0.7342
Comprehensive and Collaborative Strategies	0.74	[0.38, 1.45]	0.3764
Leadership	1.08	[0.52, 2.23]	0.8442
Participation	0.85	[0.46, 1.58]	0.606
Policies, Programs and Practices	0.69	[0.33, 1.44]	0.3222

Table S7: Associations between corporate nursing home ownership and each WISH domain: sensitivity analysis comparing top to bottom quartile. Results presented show adjusted odds of being in the lowest vs. highest WISH quartile for corporate owned vs. not corporate owned nursing homes.

**Variable**	**Odds Ratio**	**95% CI**	**p-value**
Adherence	1.06	[0.61, 1.85]	0.8261
Comprehensive and Collaborative Strategies	1.11	[0.61, 2.01]	0.7333
Leadership	1.48	[0.77, 2.86]	0.2372
Participation	0.78	[0.45, 1.35]	0.3676
Policies, Programs and Practices	1.05	[0.57, 1.92]	0.8745

Adjusted for survey wave, state, number of beds, occupancy rate, percent of residents who are Medicaid recipients
